# Supplementary material for: Spin Lattice (T1) and Magnetization Transfer Saturation (MTsat) Imaging to Monitor Age-Related Differences in Skeletal Muscle Tissue
Source: Diagnostics (Basel). 2022 Feb 24;12(3):584. doi: 10.3390/diagnostics12030584 (PMC8947179; doi:10.3390/diagnostics12030584)
Supplement: Supplementary file 1 [file diagnostics-12-00584-s001.zip › diagnostics-1572677-supplementary.pdf]

diagnostics

MDPI

Supplemental Table S1: T1 values from the four sequences and MTsat values for each subject.

	T1 (secs): No Fat Suppression				
Subject	TA	MG	LG	SOL	TP
Y	1.38	1.3452	1.4481	1.4228	1.3917
Y	1.4757	1.4095	1.4619	1.3925	1.4874
Y	1.5188	1.4519	1.6573	1.4995	1.5091
Y	1.3951	1.5101	1.6237	1.4829	1.4506
Y	1.2951	1.4692	1.6133	1.4194	1.4153
Y	1.6271	1.4167	1.5886	1.4988	1.5399
Y	1.5298	1.4883	1.696	1.5051	1.4822
Y	1.4852	1.5179	1.6488	1.4731	1.4822
Y	1.4052	1.4335	1.5361	1.4283	1.427
Y	1.5553	1.6632	1.6344	1.5061	1.5922
S	1.4761	1.5908	1.5084	1.4728	1.4773
S	1.50121	1.5696	1.6784	1.427	1.4162
S	1.4907	1.5841	1.321	1.3666	1.5428
S	1.6405	1.4963	1.604	1.4163	1.3992
S	1.4535	1.3512	1.488	1.4971	1.4971
S	1.565	1.5881	1.6548	1.5777	1.5453
S	1.6073	1.3536	1.6006	1.3698	1.4777

	T1 (secs):Fat Saturation				
Subject	TA	MG	LG	SOL	TP
Y	0.9083	0.8473	0.9105	0.8568	0.8582
Y	1.0284	0.8674	0.8275	0.836	0.9271
Y	1.0997	0.89	1.0117	0.9197	0.9459
Y	0.9095	0.937	1.0087	0.9036	0.8983
Y	0.98	0.9171	1.0826	0.9187	0.912
Y	1.0854	0.906	0.9812	0.8907	0.9563
Y	0.9777	0.9251	0.9975	0.8927	0.8472
Y	1.0042	0.9232	1.0491	0.8927	0.9529
Y	0.9227	0.7882	0.8708	0.8792	0.8838
Y	1.0642	1.02	1.0802	0.9725	0.9887
S	1.0307	0.9724	1.085	0.9397	0.9137
S	1.043	1.0432	1.0818	0.946	0.8984
S	1.0009	0.9718	0.949	0.8863	0.9091
S	1.0828	0.9817	1.046	0.9038	0.8932
S	0.9288	0.9121	1.047	1.0995	0.9051
S	1.0275	0.9889	0.8525	0.9219	0.8677
S	1.1836	0.843	0.9773	0.8834	0.9054

	T1 (secs): Water Exc (1:1)				
Subject	TA	MG	LG	SOL	TP
Y	1.4023	1.3041	1.4144	1.3621	1.3857
Y	1.472	1.472	1.3909	1.3909	1.4893
Y	1.5165	1.4101	1.5845	1.4374	1.4713
Y	1.3546	1.454	1.5535	1.4621	1.4237
Y	1.4489	1.4295	1.5579	1.3932	1.4146
Y	1.6114	1.4047	1.5374	1.4292	1.4724
Y	1.481	1.5043	1.5923	1.4386	1.4384
Y	1.4199	1.4664	1.6293	1.4311	1.4489
Y	1.3843	1.3631	1.4793	1.3925	1.3846
Y	1.5458	1.5835	1.5424	1.495	1.5061
S	1.5136	1.5458	1.5893	1.4278	1.4867
S	1.5191	1.523	1.6715	1.4198	1.3894
S	1.4892	1.5661	1.4646	1.4202	1.4747
S	1.5768	1.5205	1.6104	1.4446	1.3981
S	1.3753	1.4056	1.5166	1.5032	1.4003
S	1.513	1.5178	1.5188	1.4954	1.4246
S	1.5577	1.3384	1.5841	1.4001	1.4345

	T1 (secs): Water Exc (1:2:1)				
Subject	TA	MG	LG	SOL	TP
Y	1.4378	1.3026	1.4315	1.3769	1.3806
Y	1.4632	1.3628	1.3993	1.3627	1.4662
Y	1.5274	1.3883	1.5437	1.434	1.4615
Y	1.3041	1.412	1.5035	1.4259	1.4097
Y	1.3754	1.3992	1.5197	1.3836	1.4159
Y	1.5561	1.3855	1.5118	1.4038	1.451
Y	1.5063	1.498	1.5776	1.4355	1.4365
Y	1.4058	1.4423	1.5837	1.4235	1.4226
Y	1.3811	1.318	1.4412	1.3863	1.3746
Y	1.5366	1.5531	1.4784	1.4618	1.506
S	1.4772	1.5658	1.6047	1.5104	1.4145
S	1.529	1.435	1.6345	1.4238	1.3985
S	1.7368	1.8419	1.7541	1.6869	1.7408
S	1.5484	1.5213	1.5924	1.4045	1.3909
S	1.3663	1.4349	1.4997	1.4539	1.3837
S	1.4928	1.4863	1.4771	1.4915	1.403
S	1.6488	1.3335	1.599	1.4117	1.4327

	MTsat (%)				
Subject	TA	MG	LG	SOL	TP
Y	3.352	3.5213	3.4969	3.6556	3.5334
Y	3.1977	3.498	3.498	3.498	3.457
Y	3.1602	3.5351	3.4874	3.6167	3.3729
Y	3.153	3.4032	3.4947	3.5386	3.4089
Y	3.2092	3.4516	3.4516	3.4326	3.3741
Y	3.1074	3.3594	3.2956	3.5587	3.4137
Y	3.1074	3.3594	3.2956	3.5587	3.4137
Y	3.3125	3.5876	3.1622	3.5619	3.4416
Y	3.3042	3.799	3.5403	3.5118	3.5206
Y	3.0039	3.1642	3.1012	3.3582	3.1145
S	3.2177	3.2815	2.9717	3.2117	2.9717
S	3.1581	3.2145	2.8927	3.139	3.3046
S	3.2223	3.395	3.2327	3.5295	3.3231
S	3.1766	3.166	3.0964	3.5669	3.5558
S	3.1722	3.3869	3.1177	3.3288	3.3491
S	3.0008	3.1547	3.3875	3.1896	3.4376
S	3.158	3.5597	3.1774	3.565	3.4316

Medial gastrocnemius (MG), lateral gastrocnemius (LG), sol (SOL), tibialis posterior (TP), tibialis anterior (TA); Young (Y) and Senior (S) subjects. The T1 and MTsat values are computed in ROIs as shown in Figure 1.
